# Removal of port-site and pelvic parasitic fibroids after laparoscopic myomectomy with morcellation: a case report and literature review

**DOI:** 10.3389/fmed.2025.1563914

**Published:** 2025-10-14

**Authors:** Yushi Wu, Yi Dai, Xiaoyan Li, Jinghua Shi, Zhiyue Gu, Chenyu Zhang, Hailan Yan, Jinhua Leng

**Affiliations:** ^1^Department of Obstetrics and Gynecology, Peking Union Medical College Hospital, Chinese Academy of Medical Science & Peking Union Medical College, Beijing, China; ^2^National Clinical Research Center for Obstetrics & Gynecologic Diseases, Beijing, China

**Keywords:** parasitic leiomyoma, laparoscopic myomectomy, surgery, morcellation, case report

## Abstract

We present the case of a 39-year-old Chinese woman who presented with a palpable mass in her abdominal wall and pelvic cavity 9 years after undergoing laparoscopic myomectomy. Six abdominal wall fibroids, 18 intramural fibroids, 1 posterior adenomyoma, and 6 uterine rectal fibroids were excised. Abdominal wall fibroids were located near the right trocar site of the previous laparoscopic myomectomy, with a total diameter of 15 cm. Histopathologic examination confirmed leiomyoma. Port-site PL is a rare complication caused by power morcellation following laparoscopic surgery. Even rarer are cases involving both the abdominal wall and the pelvic cavity. To reduce the occurrence of port-site PL, it is crucial to prevent tissue dissemination via excessive fragmentation. In addition to exploring and irrigating pelvic and abdominal cavities, diligent efforts should be made to irrigate the trocar-site abdominal wall to prevent tissue implantation. It is recommended that morcellation be performed, whenever feasible, within a containment bag or by conducting intracapsular myomectomy.

## Introduction

Parasitic leiomyoma (PL) refers to the implantation of uterine fibroids outside the uterus (1). It can be spontaneous, potentially arising from the detachment of pedunculated sub-serosal leiomyoma, and iatrogenic, caused by non-malignant sequelae of tissue disseminated through power morcellation following laparoscopic surgery, receiving blood from other organs (2, 3). With the increasingly widespread application of power morcellation, a series of cases of this rare condition have been reported in recent years.

In this study, we report the case of a 39-year-old woman with PL in the right lower abdominal wall and uterine rectal fossa following laparoscopic myomectomy with power morcellation. Furthermore, we review the current literature on iatrogenic PL.

## Case description

We present the case of a 39-year-old Chinese woman who presented to our hospital with the progressive enlargement of a painless lump in the right lower abdominal wall. It was revealed that her condition had persisted for more than 8 years. She had undergone laparoscopic myomectomy 9 years earlier for the removal of uterine fibroids, during which an intramural fibroid with a diameter measuring >7 cm was excised. The fibroid was morcellated using a power morcellation system without in-bag containment. The excised tissues were removed through the trocar in the right lower abdomen.

One year after the surgery, the patient noticed a mass in the right lower quadrant of the abdomen through self-palpation. Measuring 2 cm in diameter on ultrasound examination, the mass exhibited gradual enlargement during irregular follow-up visits.

The patient denied experiencing symptoms such as increased menstrual flow, prolonged menstrual period, skin ulceration, pigmentation, and other discomforts. She did not accept any treatment during the course of her condition. Her only childbirth was through vaginal delivery, which had happened before the initial surgery. Previously, she had dysmenorrhea with a VAS score of 4–5 and exhibited no urinary or bowel symptoms.

The results of preoperative gynecological examination revealed an anterior abdominal wall mass measuring 12–14 cm in length, located in the right lower abdomen. The abdominal wall mass, which was firm, movable, and non-tender with a distinct boundary, was located at the previous port site used for morcellation and tissue extraction during myomectomy. Furthermore, her uterus appeared to be enlarged.

**Figure 1:**
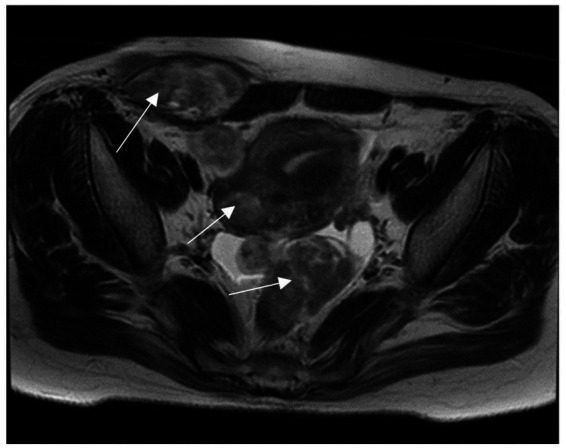
MRI shows the right anterior abdominal wall, myometrium, and multiple pelvic masses.

Ultrasonography revealed a uterus measuring 6.1 × 7.0 × 5.0 cm, exhibiting multiple fibroids and adenomyosis, with the largest fibroid, measuring 4.7 × 3.7 cm, located in the posterior uterine wall. The right lower abdominal wall revealed multiple hypoechoic regions beneath the skin. Some of these regions were in close proximity to the uterus, with the largest measuring 7.7 × 7.3 × 2.8 cm. Abdominopelvic magnetic resonance imaging (MRI) showed multiple masses in the anterior abdomen, myometrium, and lower pelvic cavity, ranging from 2.0 to 6.8 cm in length (Figure 1). A complete blood test revealed a hemoglobin level of 11.8 g/dL and a serum CA125 level of 36.3 U/mL.

**Figure 2:**
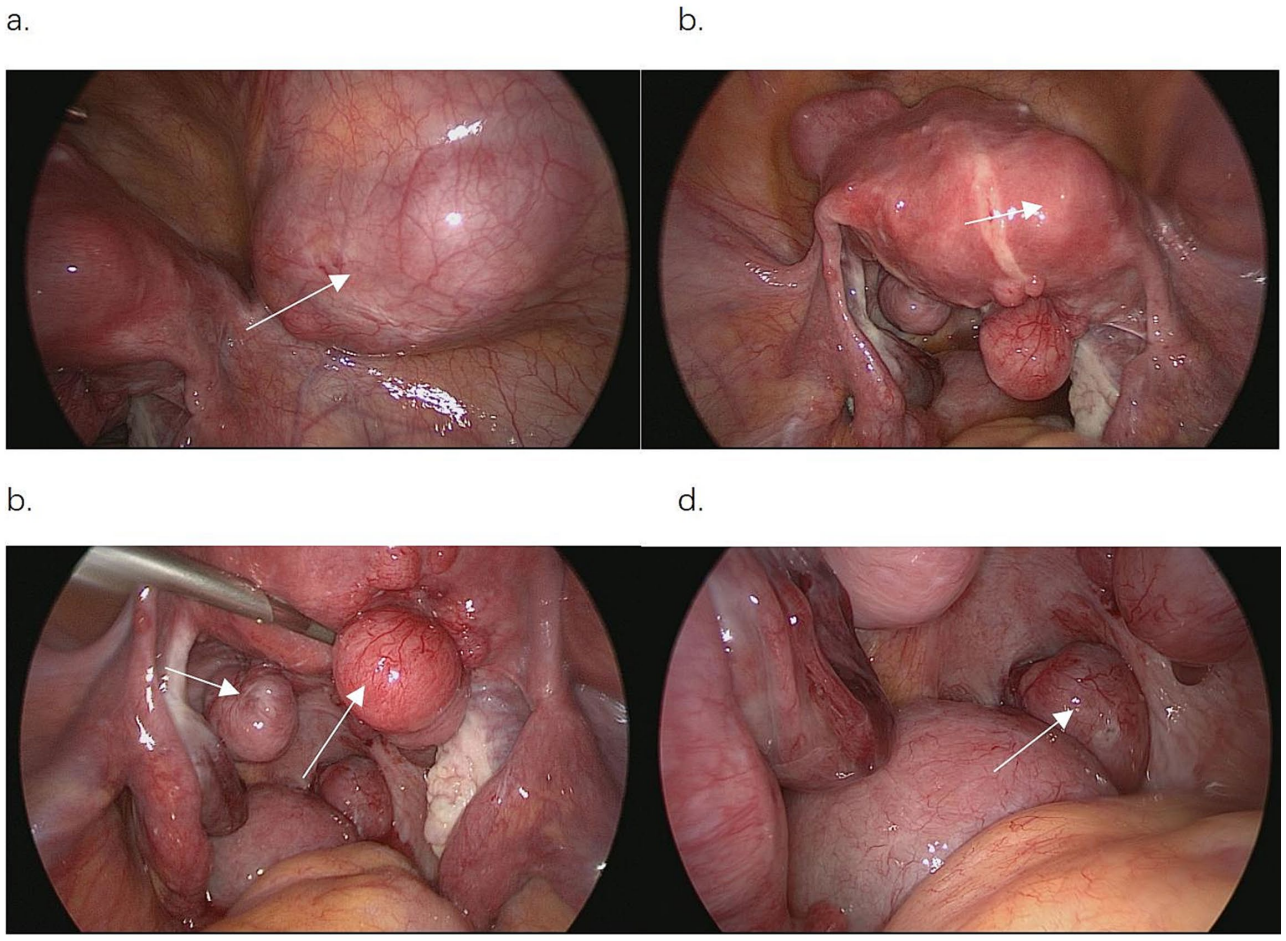
Abdominal wall and pelvic parasitic leiomyomas under laparoscopic detection. **(a)** Parasitic myomas in the abdomen and protruding into the pelvic cavity. **(b)** The uterus was altered after myomectomy, and there were multiple masses in the pelvis and uterus. **(c)** Multiple fibroid nodules in the pelvic cavity. **(d)** Largest pelvic mass, implanted in the anterior wall of the rectum.

Management options were discussed with the patient, who opted for laparoscopic exploration. Pelvic exploration showed an enlarged uterus equivalent to 12 weeks of pregnancy. Multiple subserosal and intramural fibroids were observed, with significant thickening of the posterior myometrium. Both ovaries and fallopian tubes appeared to be normal. In the uterine rectal fossa, six fibroid nodules, each with a diameter ranging from 1 to 8 cm, were identified, and the largest nodule was implanted in the anterior wall of the rectum, involving the seromuscular layer. An abdominal wall nodule, ~5 cm in diameter, protruded into the pelvic cavity with the peritoneum intact (Figure 2). Furthermore, an 8-cm-diameter fibroid nodule was palpable in the right lower abdominal wall, showing clear borders and acceptable mobility. Because of the impossibility of removing all of the fibroids using laparoscopic surgery, the procedure was converted to an open laparotomy.

We performed a suprapubic incision of >10 cm to meticulously explore the pelvic cavity and retrieve all of the parasitic myomas. During the surgery, both the abdominal wall mass and the pelvic mass were successfully removed intact. Uterine fibroids were also excised, and the adenomyosis lesion was excised in the shape of a wedge.

**Figure 3:**
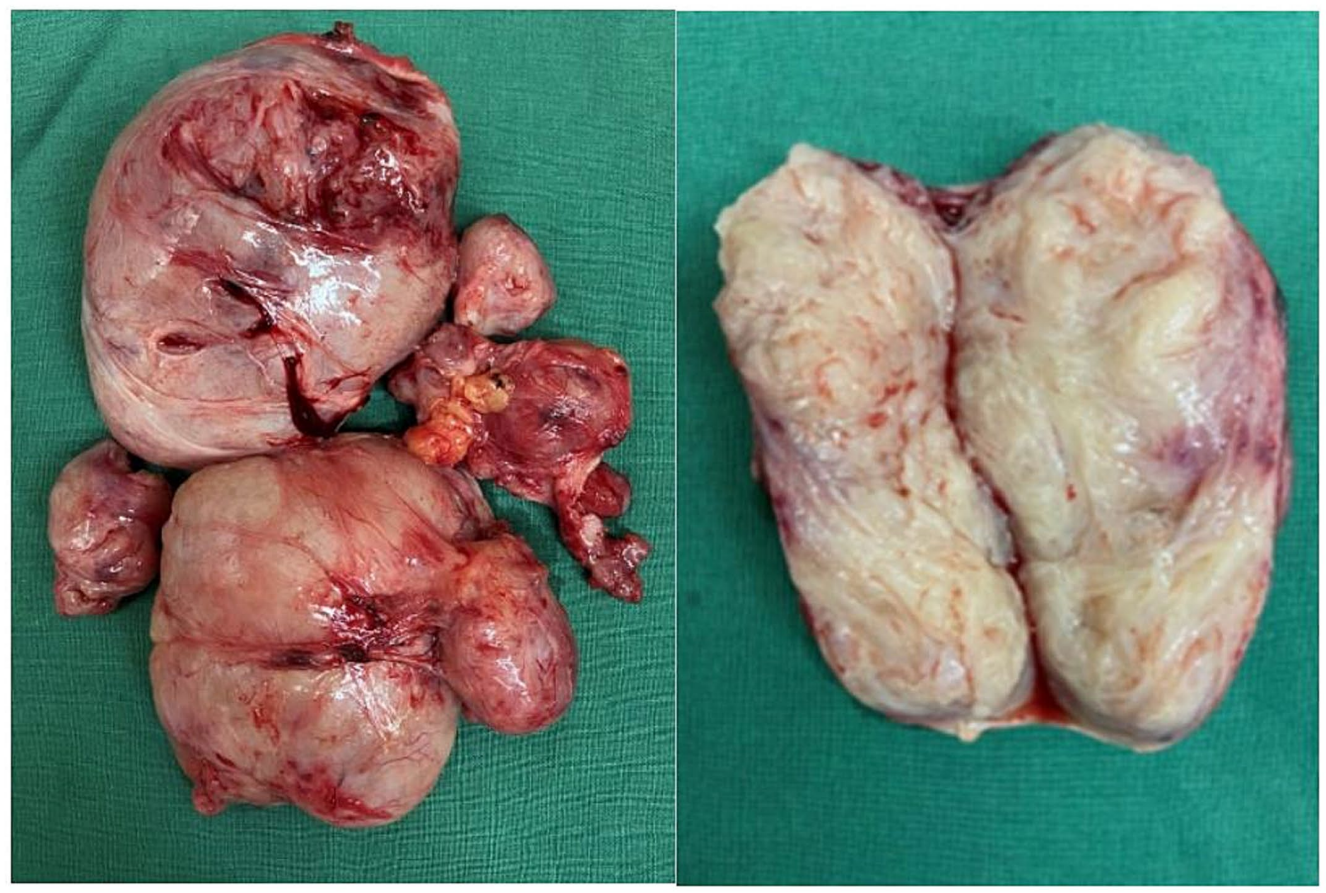
Parasitic myoma after excision.

Fibroid nodules, dissected under the stage, appeared to be soft and degenerate (Figure 3). In total, 6 abdominal wall fibroids, 18 intramural fibroids, posterior adenomyomas, and 6 uterine rectal fibroids were excised. The intraoperative blood loss was ~200 mL.

Histopathological examination of the abdominal wall mass showed features consistent with uterine fibroids and cellular richness in some regions. The uterine masses revealed characteristics consistent with both fibroids and adenomyomas. Furthermore, the mass in the uterine rectal fossa had interlacing bundles of benign smooth muscle fibers, which accorded with leiomyoma. The patient experienced a trouble-free postoperative recovery. Informed consent was obtained from the patient herself for this case report.

## Discussion

PL, which refers to the ectopic implantation of uterine fibroids, establishes vascular connections with adjacent structures and the peritoneum, enabling its survival and growth (1). The International Federation of Gynecology and Obstetrics has classified PL as type 8 (4). PL can occur spontaneously, probably originating from the detachment of pedunculated subserosal leiomyomas, or it can be iatrogenic, such as when it is induced by the inadvertent dissemination of tissue fragments during power morcellation through laparoscopic surgery (2, 3). While PL was initially described in 1909, it occurs relatively rarely. However, with the increasing availability of laparoscopic surgery and power morcellation in recent years, a series of surgery-related PL cases have been reported. Previous studies have estimated the prevalence of iatrogenic PL at 0.07–0.95% (5–7), with the majority of patients having a history of power morcellation during surgery.

While fibroid morcellation can reduce intraoperative bleeding, shorten hospital stays, and expedite recovery times (8), it also increases the risk of occult uterine sarcoma dissemination. Accordingly, a safety statement issued by the U.S. Food and Drug Administration (FDA) in 2014 advised against the use of laparoscopic power morcellation for hysterectomy or laparoscopic myomectomy (9). Analogously, this caution should be taken into account during laparoscopic procedures for the extraction of non-malignant uterine tissue. A paramount concern during fibroid management is the inadvertent morcellation of an undiagnosed uterine sarcoma, which carries significant prognostic implications. The preoperative distinction between benign leiomyomas and sarcomas remains challenging, as imaging and clinical features often overlap. Recent advances focus on improving risk stratification and diagnostic precision. Liquid biopsy analysis of circulating tumor DNA, novel MRI radiomics features, and molecular biomarkers shows promise in identifying high-risk lesions preoperatively. As highlighted by recent research (10), integrating these model-based approaches could mitigate the risk of sarcoma dissemination during minimally invasive surgery, enabling more tailored patient counseling and procedural planning.

In this case, the patient was found to have an abdominal wall mass 1 year postoperatively, which was later diagnosed as a port-site implantation of PL. Afterward, she developed intrapelvic PL, and the uterine fibroids recurred. As a rare complication of laparoscopic myomectomy, PL is generally reported to occur as an implant isolated within the pelvic cavity or abdominal wall. Cases involving both the abdominal wall and the pelvic cavity concurrently are even rarer. To date, there have been only three similar reported cases (Table 1), and the case reported in this study represented the largest diameter of abdominal-wall PL.

**Table 1:** Literature report PL cases involving the abdominal wall and pelvis.

Study	Year	Age	First operation	Morcellation	Second operation	Number of masses	Size	Location	Pathology	Prognosis
Paul and Koshy (14)	2006	28	LM	Yes	LM	3	2	Port-site, fundus, right paracolic gutter	Leiomyoma	–
Kriplani I (15)	2023	28	LM	Yes	LM	26	–	Ilium, transverse, descending, and sigmoid colon, rectum, left tube, left ovary, pouch of Douglas, bilateral uterosacrals, uterovesical fold, and anterior abdominal wall	Leiomyoma	No recurrence of PL until 6 months postoperative.
Kai (16)	2020	30	LM	Yes	LM	17	3	Intramurals, abdominal wall, and pouch of Douglas	Leiomyoma	No recurrence of PL until 17 months postoperative.

This case illustrates that PL can simultaneously develop both in the pelvic cavity and in the abdominal wall, displaying pathological features that are distinct from those of eutopic leiomyomas.

According to previous research, iatrogenic PL is the result of non-malignant sequelae caused by tissue dissemination after laparoscopic surgery (3). The case reported in this study supports this view. The abdominal wall is anatomically shielded by the parietal peritoneum. Laparoscopic exploration revealed the peritoneum to be intact, and the pelvic mass was isolated from the abdominal wall mass. Thus, it was concluded that the abdominal wall mass did not result from invasion by the pelvic mass, but rather from the previous implantation of fibroid fragments in the abdominal wall. As dynamic morcellation produces small fragments, there is an increased risk of residual fragments and subsequent implantation. The abdominal wall mass in this case was located at the trocar site of the previous myomectomy, and this speculation resulted from an attempt to remove the fibroid fragment along with the cannula. If all of the fibroid tissue was not completely contained within the cannula, fragments could have remained in the trocar track. This clarifies why it is important for surgeons to avoid removing fragments without protective measures. Small fragments can be safely retrieved using protective measures, while larger specimens need to be cut into smaller pieces before they can be retrieved. In cases of suspected malignancy, tissue should be completely excised through an enlarged incision or minilaparotomy. Moreover, in addition to ensuring adequate irrigation and meticulous inspection of the pelvis, it is essential to rinse the abdominal wall at the trocar site to prevent the implantation of residual debris. In recent years, a number of new techniques have been introduced, among which in-bag morcellation (11) and pseudocapsular comminution of uterine fibroids (12) can mitigate the dissemination of fibroid fragments in the pelvis, thereby playing a critical role in reducing the occurrence of iatrogenic PL. The role of minimally invasive myomectomy continues to evolve amid ongoing debates regarding patient selection, long-term outcomes, and fertility implications. While laparoscopic myomectomy offers advantages in recovery time and surgical morbidity, its suitability depends on fibroid characteristics, surgical expertise, and patient priorities. Recent research emphasizes that shared decision-making should balance the benefits of approaches against specific risks (13). For complex cases, open surgery or contained morcellation within an insufflated barrier system may optimize safety. Future refinement of risk-prediction models and containment technologies will further individualize surgical planning.

## Data Availability

The original contributions presented in the study are included in the article/supplementary material, further inquiries can be directed to the corresponding author.
